# Sex moderates family history of alcohol use disorder and childhood maltreatment effects on an fMRI stop‐signal task

**DOI:** 10.1002/hbm.26221

**Published:** 2023-02-01

**Authors:** Amanda Elton, John Hunter Allen, Mya Yorke, Farhan Khan, Peng Xu, Charlotte A. Boettiger

**Affiliations:** ^1^ Department of Psychology and Neuroscience University of North Carolina Chapel Hill North Carolina USA; ^2^ Bowles Center for Alcohol Studies University of North Carolina Chapel Hill North Carolina USA; ^3^ Biomedical Research Imaging Center University of North Carolina Chapel Hill North Carolina USA

**Keywords:** alcohol use disorder, childhood adversity, early life stress, family history, fMRI, impulsivity, sex differences

## Abstract

Childhood maltreatment (CM) and a family history (FH) of alcohol use disorder (AUD) are each associated with increased impulsivity. However, their unique or shared brain targets remain unknown. Furthermore, both CM and FH demonstrate sex‐dependent effects on brain and behavior. We hypothesized that CM and FH interact in brain regions involved in impulsivity with sex‐dependent effects. 144 first‐year college students (18–19 years old) with varying experiences of CM and/or FH but without current AUD performed an fMRI stop‐signal task. We tested interactions between FH, CM, and sex on task performance and blood oxygen level‐dependent (BOLD) signal during successful inhibitions. We examined correlations between BOLD response and psychiatric symptoms. Significant three‐way interactions of FH, CM, and sex were detected for brain and behavioral data, largely driven by male subjects. In males, CM was associated with poorer response inhibition but only for those with less FH; males with higher levels of both CM and FH demonstrated better response inhibition. Three‐way interaction effects on voxel‐wise BOLD response during response inhibition were found in bilateral middle frontal gyrus, left inferior frontal gyrus, dorsomedial prefrontal cortex, and posterior cingulate cortex. Network‐level analyses implicated the left frontoparietal network, executive control network, and default‐mode network. Greater BOLD response in these networks correlated with lower depressive, impulsive, and attentional symptoms, reduced alcohol misuse, greater resilience scores, and heightened trait anxiety. The results highlight sex‐divergent effects of heritable and environmental risk factors that may account for sex‐dependent expression of psychopathology in response to risk factors.

## Graphical abstract

Significant three‐way interactions of family history of alcohol use disorder, childhood maltreatment, and sex were detected for brain and behavioral measures of response inhibition, largely driven by male subjects. Males with the greatest exposure to risk factors exhibited preserved response inhibition and enhanced frontoparietal BOLD responses. Greater BOLD response in these regions was associated with reduced psychopathology and greater self‐reported resilience.

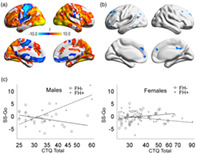

## 1 INTRODUCTION

Alcohol use disorder (AUD) affects approximately 10% of young adults (SAMHSA, 2019) and is characterized by a loss of control over the use of alcohol. Risk for AUD is related to both environmental and heritable factors. For example, early life adversity is associated with a dose‐related increased risk for alcohol misuse and addiction (Anda et al., 2002; Dube et al., 2002; Dube et al., 2006; Enoch, 2011; Felitti et al., 1998). On the other hand, approximately half of the risk for AUD is heritable (Agrawal & Lynskey, 2008; Kendler, Aggen, et al., 2012), and a family history (FH) of addiction is especially associated with risk for AUD (Anda et al., 2002; Kendler, Sundquist, et al., 2012), independent of the adversity related to parental AUD (Anda et al., 2002). Substantial evidence further suggests that early life adversity and heritable risk interact to confer vulnerability for AUD. For example, synergistic effects of early life adversity and FH have been found in large samples, indicating that these risk factors produce supra‐additive risk for AUD (Fenton et al., 2012; Kendler, Sundquist, et al., 2012). Similarly, synergistic effects of early life adversity and certain genetic polymorphisms on addiction risk have also been demonstrated (Blomeyer et al., 2008; Covault et al., 2007; Enoch, 2012; Enoch et al., 2010; Jabbi et al., 2007; Kim‐Cohen et al., 2006; Laucht et al., 2012; Nelson et al., 2010; Ray et al., 2013; Schellekens et al., 2013; Schmid et al., 2010). However, despite the clear relationships between both early life adversity, heritable risk factors, and their interaction on AUD, the neural and behavioral mechanisms by which these two forms of risk contribute to the development of addiction are incompletely determined.

One mechanism by which risk factors may confer vulnerability for AUD is through increased impulsivity. This broad construct is used to describe multiple facets of behavior, including sensation seeking, reduced future‐oriented thinking, lack of perseverance, and poor motor response inhibition (Dick et al., 2010). The clinical criteria for AUD strongly implicate forms of impulsivity as an underlying behavioral mechanism. In fact, individuals with AUD demonstrate increased impulsivity on tasks that measure response inhibition, such as stop‐signal tasks (Lawrence et al., 2009; Sjoerds et al., 2014). Furthermore, impulsive behavior in childhood and adolescence, prior to significant substance use, also predicts later life alcohol use (Mahmood et al., 2013; Nigg et al., 2006; Wong et al., 2006), suggesting these behaviors precede the development of AUD and represent a neurobehavioral mechanism of risk.

Indeed, individuals with risk factors for AUD demonstrate poorer performance on laboratory tasks of impulsivity compared with low‐risk individuals. For example, children and young adults at risk for AUD based on FH demonstrate increased impulsivity on a response inhibition task compared with individuals without this risk factor (Acheson et al., 2011; Dougherty et al., 2014). Adolescents with FH also exhibit altered trajectories of response inhibition neurocircuitry, including middle cingulate, caudate, and middle frontal gyrus, indicating brain mechanisms through which FH might promote risk for AUD (Schweinsburg et al., 2004). A history of CM also associates with more impulsive responding and functional alterations in particular brain regions. For example, adolescents exposed to childhood neglect show deficits in response inhibition related to increased neural activity in the anterior cingulate cortex, inferior frontal cortex, posterior insula, and striatum relative to low‐risk controls (Mueller et al., 2010). Furthermore, individuals exposed to sexual assaultive trauma in childhood have atypical electrophysiologic developmental trajectories of frontal theta during response inhibition, which associates with increased risk for AUD symptoms (Meyers et al., 2019). Additionally, during a stop‐signal task, adults with a history of abuse and neglect display altered functional connectivity within an inhibitory control network involving the anterior cingulate cortex and inferior frontal cortex (Elton et al., 2014). These studies provide emerging evidence for an association between environmental and heritable AUD risk factors and deficits in response inhibition related to alterations in prefrontal circuit function.

Biological sex also significantly affects the relationship between AUD risk factors, impulsivity, and AUD. Not only are there sex‐related variations in the healthy brain (Cahill, 2006), including functional differences during response inhibition (C‐sR et al., 2006), but there are also sex differences in the neurodevelopmental effects of traumatic experiences (the rates of which differ between the sexes; Sedlak & Broadhurst, 1996) on the neural substrates of response inhibition (Elton et al., 2014). For example, childhood maltreatment‐related alterations in the functional connectivity strength and organization of a neural network implicated in inhibitory control are sexually dimorphic, such that more left (vs. right)‐lateralized network connectivity impaired response inhibition in males but improved response inhibition in females (Elton et al., 2014). Genetic risk for AUD may also vary by sex, perhaps due to sex differences in etiological pathways or patterns of transmission (Prescott, 2002). Furthermore, studies of healthy adults with FH demonstrate significant sex‐by‐risk interactions on both brain (DeVito et al., 2013) and behavioral (Weafer et al., 2015) measures of response inhibition. Differential effects of risk factors in males and females could partly account for sex differences in the expression of AUD and other psychiatric disorders.

Despite mounting evidence that heritable and environmental factors each influence response inhibition behavior and associated neurocircuitry, neuroimaging studies have yet to examine their unique and combined influence on the brain within a single sample. Investigating both factors in a single study is especially important to disentangle their effects since FH, a proxy for heritable risk, is often associated with CM (Anda et al., 2002). Furthermore, we sought to examine how effects of risk factors differ by sex and potentially relate to sex differences in the expression of psychiatric symptoms. We hypothesized that greater levels of each risk factor would be associated with reduced inhibitory control, sex‐specific patterns of diminished activation in prefrontal brain regions, and their combination would result in supra‐additive impacts on brain and behavioral markers of impulsivity. We tested these hypotheses in a sample of 144 male and female first‐year college students with varying levels of heritable and environmental risk for AUD. Subjects performed a stop‐signal task while undergoing fMRI. Although risk factors were associated with hypothesized reductions in inhibitory control and attenuated neural responses in a subset of at‐risk subjects, our data also revealed unexpected sex‐dependent brain functional adaptations.

## 2 METHODS

### 2.1 Subjects

We recruited 165 first‐year college students for a neuroimaging study with longitudinal follow‐up surveys. The study was approved by the University of North Carolina (UNC) Office of Human Research Ethics and participants gave written informed consent to participate. All participants were 18–19 years of age and in their first year in a 4‐year undergraduate degree program at UNC (*n* = 140) or other colleges in the surrounding region including Duke University (*n* = 12), North Carolina State University (*n* = 6), Elon University (*n* = 3), North Carolina Central University (*n* = 2), East Carolina University (*n* = 1), and Highpoint University (*n* = 1). Potential subjects were excluded for MRI contraindications such as claustrophobia and nonremovable metal in the body, as well as left‐handedness, psychoactive medication or other routine drug use, neurological disorders, and psychiatric disorders. Lifetime mood or anxiety disorders without meeting criteria currently were not excluded. The Mini‐International Neuropsychiatric Interview (M.I.N.I.) for DSM‐IV (Sheehan et al., 1998) assessed for the presence of psychiatric disorders, whereas DSM‐5 criteria was used to assess current or lifetime AUD or substance use disorder. No participants tested positive on a urine drug screen (Biotechnostix, Inc., Markham, ON) for recreational substance use (including cocaine, cannabis, opioids, amphetamines, methamphetamine) on the day of the MRI scan. Alcohol breathalyzer tests (FC‐10, Lifeloc Inc., Wheat Ridge, CO) were similarly negative. 21 subjects had incomplete fMRI or behavioral stop‐signal task data and were excluded from the reported analyses, providing a final sample size of 144 subjects (95 females). To explore the possibility that BOLD response during the stop‐signal task would predict future substance use, we also sent follow‐up surveys 1 year following the fMRI scan to 140 subjects who consented to participate in follow‐up procedures. The follow‐up surveys assessed recent substance use and other measures of mental health (see *Self‐Report Instruments*). One‐year follow‐up data was available for 108 subjects (76 females).

### 2.2 Self‐report instruments

Self‐report questionnaires were administered via Research Electronic DATA Capture (REDCap; Harris et al., 2009) surveys sent to subjects via email. FH was assessed with the Family History Assessment Module (FHAM; Rice et al., 1995). A FH density score was calculated from the total prevalence of AUD among biological parents and second‐degree relatives with AUD previously published methodology (Cservenka & Nagel, 2012). The score is a weighted total of relatives with an AUD, where affected parents are weighted 0.5, grandparents are weighted 0.25, and maternal and paternal aunts and uncles are each weighted 0.25 divided by the total number of aunts and uncles on that side of the family.

Childhood maltreatment was measured with the Childhood Trauma Questionnaire (CTQ; Bernstein et al., 2003). The CTQ includes subscales pertaining to physical, emotional, and sexual abuse, as well as physical and emotional neglect. A log‐transformed total score of all five subscales was used in primary analyses. Secondary analyses explored effects of individual subscales.

Baseline surveys also measured ADHD‐related hyperactivity and inattention (Conners Adult ADHD Rating Scale [CDDR]; Conners et al., 1998), psychological resilience (Connor‐Davidson Resilience Scale [CD‐RISC‐10]; Connor & Davidson, 2003), and impulsivity (UPPS‐P short version (Cyders et al., 2014) and Barratt Impulsiveness Scale [BIS]; Patton et al., 1995). Both baseline and 1‐year follow‐up surveys included self‐report measures of depression (Beck Depression Inventory [BDI]; Beck et al., 1961), anxiety (State–Trait Anxiety Inventory [STAI]; Spielberger et al., 1980), alcohol misuse (Alcohol Use Disorders Identification Test [AUDIT]; Saunders et al., 1993), and other substance use including cannabis and tobacco (Customary Drinking and Drug Use Record [CDDR]; Brown et al., 1998).

### 2.3 fMRI

FMRI data during the stop signal task were acquired with a Siemens 3 T Prisma scanner with a 32‐channel TEM send‐receive radio frequency (RF) head coil (Siemens Healthineers, Erlangen, Germany). Blood oxygenation level‐dependent (BOLD) images were acquired in sagittal orientation using a multiband echo‐planar imaging (EPI) sequence with multiband factor = 8, TR = 800 ms, TE = 37 ms, flip angle = 52°, 2 mm isotropic voxels, 72 slices, field of view (FOV) = 208 × 208, bandwidth = 2290 Hz/pixel, interleaved acquisition. Due to a scanner update, we worked with an MRI physicist to adjust the sequence for the final 27 subjects to bandwidth = 2186 Hz/pixel and TE = 38.2 ms. These changes have smaller effects on the BOLD contrast than are typically seen in test–retest scans with the same sequence. Thus, the update negligibly impacted the BOLD signal. We used an anterior‐to‐posterior (AP) phase encoding direction for the first 413 TRs and posterior‐to‐anterior (PA) phase encoding for final 413 TRs. The second scan started upon the completion of the first scan, providing a continuous scan of approximately 11 min.

High‐resolution magnetization‐prepared rapid gradient‐echo (MPRAGE) T1‐weighted parameters were: TR = 2530 ms, TE = 2.3 ms, flip angle = 9°, 1 mm isotropic voxels, 176 sagittal slices, FOV = 256 × 256.

### 2.4 Stop signal task

The stop signal task was presented with Psychopy software, and consisted of 300 trials, divided into two runs corresponding with the AP and PA phase encoding directions of the fMRI scan. Within each run were three 100 s blocks of 50 trials each flanked by four 12 s blocks of fixation. There was a 2000 ms fixed interval between trials. At the start of each trial, an arrow appeared in the center of the monitor with an equal probability of facing left or right. Subjects were instructed to make a left or right button press corresponding with the arrow direction. In 75 trials (25%), a stop signal, an “X”, appeared overtop the arrow after a brief delay, the stop‐signal delay (SSD). Subjects were instructed to attempt to withhold their response if the X appeared. The SSD started at 200 ms and was adaptively lengthened by 50 ms after each successful stop and shortened by 50 ms after a failure to withhold a response. The SSD was restricted to a minimum of 50 ms and a maximum of 900 ms. Twenty‐five trials (8%) were “null” trials consisting of a bidirectional arrow visual stimulus to aid in deconvolving BOLD signals. Trial order was pseudorandomized. Speed and accuracy were equally emphasized, and subjects were reassured that it was normal to not be able to inhibit their response on every stop trial. Subjects underwent a 50‐trial practice prior to entering the scanner, and subjects were reminded to not wait for the stop signal.

### 2.5 Behavioral analysis

Stop‐signal reaction time (SSRT) was calculated using published methods (Congdon et al., 2012; Elton et al., 2014). Specifically, we subtracted the mean stop‐signal delay from the *n*th percentile go trial response time, where the *n*th percentile corresponds with successful stop rate. We adjusted the successful stop rate for variation in the go rate (i.e., go trials without misses) as described previously (Elton et al., 2014) to account for successful inhibitions that may have resulted from general non‐responding during the task. We applied lenient criteria for identification of outlier subjects based on published recommendation (inhibition rate on stop trials outside 25–75%, go rate < 60%, go accuracy<90%, SSRT<50 ms; Congdon et al., 2012), which resulted in the exclusion of one subject with a negative SSRT. Despite explicit instructions, numerous subjects displayed evidence of employing waiting strategies. Because the longest SSD included in the task was 900 ms, we further excluded 20 subjects (from behavioral analyses only) with prolonged response times that averaged >950 ms (maximum SSD + lower limit SSRT).

We tested effects of FH, CM, and sex, as well as their interactions, on SSRT in linear regression models. Model 1 simultaneously tested effects of FH and effects of CM, controlling for sex. Model 2 tested interacting effects of FH and CM, controlling for sex. Model 3 testing interacting effects of FH and sex, as well as interacting effects of CM and sex, within the same model. Model 4 tested the three‐way interaction of FH, CM, and sex.

### 2.6 fMRI preprocessing

Data were preprocessed with fMRIPrep (Esteban et al., 2019). The T1‐weighted (T1w) image was corrected for intensity non‐uniformity (INU) with *N4BiasFieldCorrection* in ANTs 2.3.3(Avants et al., 2011). The T1w‐reference was then skull‐stripped with a *Nipype* implementation of *antsBrainExtraction.sh* workflow (from ANTs), using OASIS30ANTsas target template. Brain tissue segmentation of cerebrospinal fluid (CSF), white‐matter (WM) and gray‐matter (GM) was performed *recon‐all* (FreeSurfer 6.0.1; Fischl, 2012), and the brain mask estimated using *fast* (FSL 5.0.9; Jenkinson et al., 2012). Brain surfaces were reconstructed using *recon‐all*, and the brain mask estimated previously was refined with a custom variation of the method to reconcile ANTs‐derived and FreeSurfer‐derived segmentations of the cortical gray‐matter of Mindboggle. Volume‐based spatial normalization to one standard space (MNI152NLin2009cAsym) was performed through nonlinear registration with *antsRegistration*, using brain‐extracted versions of both T1w reference and the T1w template (i.e., ICBM 152 Nonlinear Asymmetrical template version 2009c).

BOLD preprocessing included motion estimation and realignment, slice time correction, distortion correction, registration to the T1 image, normalization to Montreal Neurological Institute (MNI) standard space (MNI152NLin2009cAsym), and confounds estimation. First, a reference volume and its skull‐stripped version were generated using a custom methodology of fMRIPrep. A B0‐nonuniformity map was estimated based on two echo‐planar EPI references with opposing phase‐encoding directions, with *3dQwarp* (Analysis of Functional Neuroimages [AFNI]; Cox, 1996). Based on the estimated susceptibility distortion, a corrected EPI reference was calculated for a more accurate co‐registration with the anatomical reference. The BOLD reference was then co‐registered to the T1w reference using *bbregister* (FreeSurfer) which implements boundary‐based registration. Co‐registration was configured with six degrees of freedom. Head‐motion parameters with respect to the BOLD reference (transformation matrices, and six corresponding rotation and translation parameters) are estimated before any spatiotemporal filtering using *mcflirt* (FSL). BOLD runs were slice‐time corrected using *3dTshift* (AFNI). The BOLD time‐series were resampled into Montreal Neurological Institute standard space (i.e., MNI152NLin2009cAsym).

### 2.7 Voxel‐wise fMRI activation analysis

Voxel‐wise activation analysis was conducted using the general linear modelling (GLM) approach (Friston et al., 1994) with 3dDeconvolve and 3dREMLfit in AFNI software (version 20.3.00). We modeled the follow trial types: successful stops, failed stops, go trials following successful stops, go trials following failed stops, go trials not following a stop trial (subsequently referred to as “Go trials”), and missed go trials. Nuisance signals were included as covariates, including 24 motion parameters (Friston et al., 1996), signals from WM and CSF voxels as well as their derivatives, squared values, and square of their derivatives, as well as the first 10 aCompCor components from the combined WM and CSF mask (Muschelli et al., 2014). Time points with a framewise displacement of >0.5 mm were censored. We calculated the contrast map of successful stops minus go trials (SS‐Go) to isolate inhibition‐related BOLD responses for each individual.

Group‐level effects of risk factors on the voxel‐wise contrast of SS‐Go were assessed with 3dMEMA in AFNI. To evaluate the independent and interacting effects of FH, CM, and sex, we tested four separate models, corresponding to Models 1–4 described in the *Behavioral Analysis* section above including three‐way interactions of FH, CM, and sex, as well as main effects and lower‐order interactions. All analyses also included Go trial response rate as a covariate since this metric is associated with a greater likelihood of successful stop trials that occur as a result of a general decrease in overall response frequency and is likely to influence the estimate of response inhibition‐related brain activation. The cluster‐size threshold required for multiple comparisons correction was calculated with 3dClustSim in AFNI after estimating the spatial autocorrelation with 3dFWHMx. Because we tested four different models, we set α = 0.0125 to correct for the increased probability of detecting significant clusters, the p‐value threshold was set to 0.005, and the calculated cluster size threshold was 72 contiguous voxels.

### 2.8 Network fMRI analyses

To reduce the number of tests performed and to aid in interpreting results, we performed analyses on large‐scale neural networks in addition to the voxel‐wise approach. Network‐level analyses were conducted using masks of 10 BrainMap networks derived from task activation analyses that were well‐matched to resting‐state networks (Smith et al., 2009) (http://www.fmrib.ox.ac.uk/datasets/brainmap+rsns/). All voxels having a z‐score value >3.0 were included in each binary mask.

To calculate network activation during response inhibition, we calculated the mean SS‐Go contrast value within each network mask for each subject. Given significant effects of Model 4 for both behavior (i.e., SSRT) and voxel‐wise maps of the SS‐Go contrast, our network‐level analyses similarly tested the three‐way interaction effects of FH, CM, and sex, covarying for go trial response rate, on SS‐Go contrast values for each network. *p* values were False Discovery Rate (FDR; Benjamini & Yekutieli, 2001) corrected for multiple tests. Supplemental analyses also explored effects of FH, CM, and sex on functional connectivity between networks using beta‐series functional connectivity analysis (Rissman et al., 2004; see Supplementary Materials).

### 2.9 Relationships between brain measures and psychiatric symptoms

To test whether variations in task‐related BOLD response associated with risk factors and sex represent negative consequences or positive adaptations, we tested the multivariate relationship between network‐level SS‐Go contrast estimates and self‐reported psychiatric symptoms. Specifically, we conducted a canonical correlation analysis in SAS (version 9.4) Proc Cancorr to identify linear combinations network‐level SS‐Go contrast estimates and psychiatric symptoms to maximize their correlation.

We included only SS‐Go activation estimates for the networks that displayed significant effects of CM, FH, and sex in the analysis described above (see *Network fMRI Analyses*). For psychiatric symptom measures, we included self‐reported symptoms reported at baseline of depression (BDI), trait anxiety (STAI), ADHD‐related hyperactivity and inattention (CAARS), and psychological resilience (CD‐RISC‐10), as well as substance use measures collected at 1‐year follow‐up, including cannabis use (0 = never used, 1 = ever used), tobacco use (0 = never used, 1 = ever used), and alcohol use (AUDIT total score). The analysis covaried for AUDIT scores at baseline (to model change in drinking), FH, CM, sex, whether baseline assessments were conducted during the Covid‐19 pandemic, and whether 1‐year follow‐up assessments were conducted during the Covid‐19 pandemic.

## 3 RESULTS

### 3.1 Demographic and questionnaire data

Characteristics of subjects included in the analyses based on self‐report questionnaires are presented in Table 1. FH density in this sample ranged from 0 to 1.5 with a median of 0.25. The mean CTQ total scores in this sample (Table 1) were somewhat higher than reported in a community sample (Scher et al., 2001), as expected due to our targeted recruitment. The sexual abuse subscale of the CTQ was higher in females based on a two‐sample t‐test, although rates were generally low (Table 1). A chi‐square test indicated that by the follow‐up, females were more likely to have used cannabis than were males. There were also sex differences in UPPS‐P first‐order factor scales. There were no other sex differences in self‐reported data.

**TABLE 1:** Behavioral and self‐report data for males and females

	Males (*n* = 49)	Females (*n* = 95)	Statistic	*p* value
Stop signal reaction time (ms)	201 ± 48	209 ± 42	*t* = −0.97	.33
Successful stop rate (%)	55.0 ± 5.2	54.4 ± 4.8	*t* = 0.71	.48
Mean stop signal delay (ms)	564 ± 187	514 ± 178	*t* = 1.55	.12
Go response rate (%)	97.1 ± 5.7	98.0 ± 5.7	*t* = −0.87	.39
Mean go trial reaction time (ms)	780 ± 19	732 ± 168	*t* = 1.53	.13
Race (%): Asian/African American or Black/ White/Native American/more than one race	35/10/42/0/13%	23/7/62/1/6%	*Χ* ^*2*^ = 6.24	.18
Ethnicity (% Hispanic/Latino)	12.5%	11.6%	*Χ* ^*2*^ = 0.03	.87
Family history density	0.29 ± 0.36	0.35 ± 0.39	*t* = −1.01	.12
Childhood Trauma Questionnaire				
Total	34.0 ± 8.6	37.1 ± 12.2	*t* = −1.77	.08
Physical abuse	6.3 ± 1.9	6.3 ± 2.7	*t* = −0.08	.94
Emotional abuse	7.9 ± 3.8	9.1 ± 4.2	*t* = −1.68	.10
Sexual abuse	5.1 ± 0.5	6.3 ± 3.1	***t* = −3.51**	**<.001**
Physical neglect	6.4 ± 2.1	6.4 ± 2.1	*t* = 0.11	.92
Emotional neglect	8.4 ± 3.4	9.1 ± 3.9	*t* = −1.11	.17
AUDIT baseline	1.9 ± 2.4	2.8 ± 2.9	*t* = −1.84	.07
AUDIT follow‐up	2.5 ± 3.1	3.7 ± 3.7	*t* = −1.53	.13
Cannabis baseline (%)	25.0%	36.7%	Χ^2^ = 1.94	.16
Cannabis use follow‐up (%)	27.1%	46.7%	**Χ** ^**2**^ **= 5.01**	**.025**
Tobacco use baseline (%)	10.4%	14.4%	Χ^2^ = 0.50	.50
Tobacco use follow‐up (%)	22.9%	17.8%	Χ^2^ = 0.53	.47
Beck depression inventory baseline	6.6 ± 7.9	8.5 ± 8.4	*t* = −1.37	.17
Connor‐Davidson Resilience Scale	29.5 ± 6.3	28.7 ± 6.0	*t* = 0.79	.43
State–trait anxiety inventory—trait anxiety	45.7 ± 4.8	46.7 ± 5.0	*t* = −1.14	.26
Conners adult ADHD rating scale				
Hyperactivity/impulsivity	6.9 ± 5.0	5.8 ± 3.8	*t* = 1.31	.20
Inattention	7.0 ± 4.7	6.4 ± 4.7	*t* = 0.50	.50
UPPS‐P				
Negative urgency	8.7 ± 3.0	8.2 ± 2.9	*t* = 1.03	.31
Positive urgency	7.9 ± 2.9	6.6 ± 2.4	***t* = 2.97**	**.004**
Sensation seeking	11.8 ± 2.6	10.5 ± 2.7	***t* = 2.72**	**.007**
Lack of premeditation	5.8 ± 1.7	6.5 ± 2.2	***t* = −2.00**	**.047**
Lack of perseverance	6.7 ± 1.7	7.3 ± 2.0	***t* = −2.09**	**.039**
Barratt impulsiveness scale				
Total	57.5 ± 9.6	56.0 ± 10.0	*t* = 0.87	.39
Attentional	15.6 ± 3.1	15.8 ± 3.8	*t* = −0.26	.79
Nonplanning	21.8 ± 4.9	20.9 ± 4.7	*t* = 1.00	.32
Motor	20.3 ± 4.2	19.3 ± 3.8	*t* = 1.43	.15

### 3.2 Behavioral data

**FIGURE 1:**
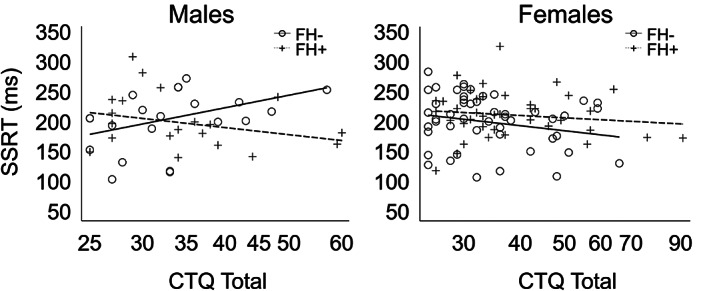
Scatter plots of the significant effects of Childhood Trauma Questionnaire (CTQ) total scores on stop signal reaction times (SSRT) by family history density (FH) and sex. For visualization purposes, FH was binarized such that subjects with a family history density ≤0.25 were designated as FH‐ and >0.25 was designated as FH+.

Stop‐signal task performance measures are presented in Table 1. A linear regression identified a significant FH × CM × sex interaction (Model 4) on SSRT (*t* = 2.88, *p* = .004). None of the other models indicated significant main or interacting effects, and neither FH nor CM showed significant independent correlational effects with SSRT (Table S1). Results of all four models are presented in Table 2. As shown in Figure 1, the plotted data indicated that in males with low levels of FH density, greater CTQ scores are associated with slower SSRTs. On the other hand, greater CTQ scores are associated with faster SSRTs in males with higher FH density. The pattern was opposite in females: Higher CTQ scores and lower FH density predicted faster SSRTs, whereas higher CTQ scores and higher FH density predicted slower SSRTs. Main effects of sex, FH, and CM and lower‐order interactions were not significant in models testing these effects.

**TABLE 2:** Results of linear regression models testing effects of family history, childhood maltreatment, and sex on stop‐signal reaction times

Model 1	Intercept	Sex	FH	CM				
*t*	5.07	0.98	1.86	−0.19				
*p*	<.001	.33	.07	.19				
Adjusted *R* ^2^ = 0.02								

Model 2	Intercept	Sex	FH	CM	FH × CM			
*t*	3.03	1.00	0.73	−0.46	−0.60			
*p*	.003	.32	.47	.65	.55			
Adjusted *R* ^2^ = 0.01								

Model 3	Intercept	Sex	FH	CM	Sex × FH	Sex × CM		
*t*	1.70	0.93	0.93	0.12	0.18	−0.86		
*p*	.09	.36	.36	.9	.86	.39		
Adjusted *R* ^2^ = 0.01								

Model 4	Intercept	Sex	FH	CM	Sex × FH	Sex × CM	FH × CM	Sex × FH × CM
*t*	−0.99	2.79	2.98	2.27	−2.94	−2.76	−2.94	2.96
*p*	.33	.006	.004	.03	.004	.007	.004	.004
Adjusted *R* ^2^ = 0.07								

*Note*: Although included in the models, lower‐order effects were not interpreted for significance.

Abbreviations: CM, childhood maltreatment measured with Childhood Trauma Questionnaire (CTQ) total scores; FH, family history density.

### 3.3 Voxel‐wise fMRI activation analysis

**FIGURE 2:**
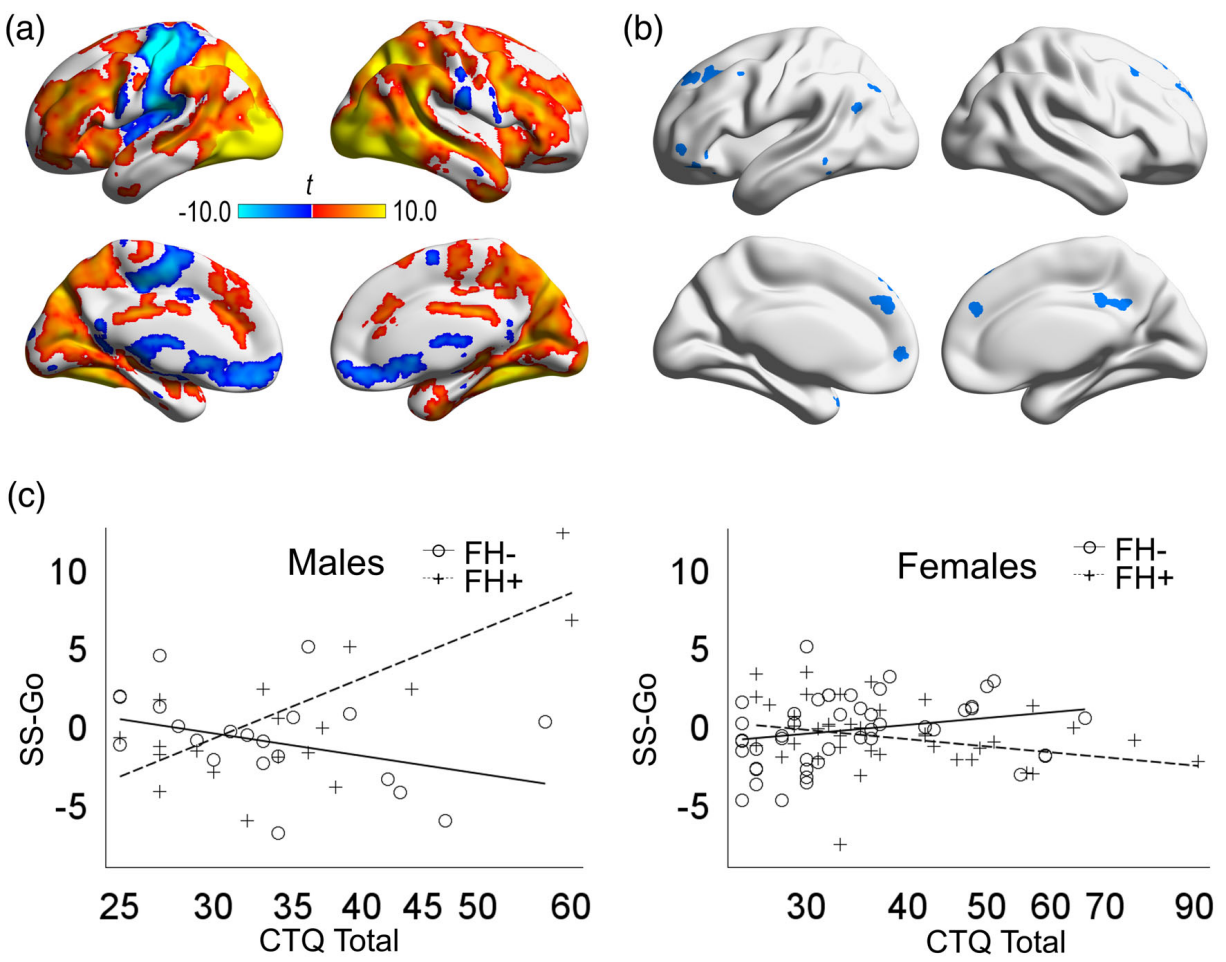
Voxel‐wise neuroimaging results. (a) Significant clusters from a one‐sample *t*‐test of the contrast between successful stop trials and go trials across all subjects. (b) Significant clusters of the three‐way interaction effect of family history density, Childhood Trauma Questionnaire (CTQ) total scores, and sex on the contrast between successful stop trials and go trials (SS‐Go). (c) Scatter plots of the effects of CTQ total scores on the SS‐Go contrast by family history density (FH) and sex on; plotted values represent the first principal component of all significant clusters. For visualization purposes, FH was binarized such that subjects with a family history density ≤0.25 were designated as FH‐ and >0.25 was designated as FH+.

Results from the voxel‐wise analysis of FH, CTQ on the SS‐Go contrast are presented in Figure 2 and Table 3. Figure 2a presents the main effect of the SS‐Go contrast in a 1‐sample test of all subjects in the study. Models 1–3 produced no significant clusters. However, Model 4, which tested the three‐way interaction of FH, CM, and sex, indicated multiple significant cortical clusters, including in medial prefrontal cortex, bilateral middle frontal gyrus, left inferior frontal gyrus, posterior cingulate cortex, and angular gyrus (Figure 2b). Scatter plots of these findings (Figure 2c) indicate that CTQ scores were associated with lesser task‐related activation (i.e., SS‐Go contrast) in males with lower FH density but greater task‐related network activation in males with higher FH density; in females, CTQ scores were associated with lesser network task‐related activation for females with higher FH density.

**TABLE 3:** Significant effects of the interaction of family history density, childhood maltreatment, and sex on the voxel‐wise contrast of successful stops minus go trials

Brain region	*n* voxels	*x*	*y*	*z*	Peak *t*‐statistic
Left inferior frontal gyrus (*Pars Orbitalis*)	389	−42.1	27.1	−19.2	−4.54
Right superior frontal gyrus	254	12.9	55	41.7	−5.65
Left superior frontal gyrus	254	−16.6	41	45	−4.05
Right middle frontal gyrus	209	41.9	20.9	52.5	−6.41
Medial prefrontal cortex	173	−2.9	44.5	32	−4.77
Left inferior temporal gyrus	129	−64.4	−51.4	−11	−4.96
Left middle frontal gyrus	127	−46.4	45.3	2.5	−4.20
Medial prefrontal cortex	122	−6.4	63	5.1	−4.37
Left angular gyrus	113	−46.9	−60.5	25.9	−4.84
Posterior cingulate cortex	109	6.2	−40.4	34.9	−4.55
Right inferior frontal gyrus (*Pars Triangularis*)	106	56.7	34.6	13.7	−5.04
Left middle frontal gyrus	90	−40.9	10.6	56.9	−4.46
Left occipital pole	88	−29.2	−99.9	−13.4	−5.80
Right cerebellum	84	21.6	−90.1	−31.4	−6.10
Right superior frontal gyrus	80	9.4	36.7	54.7	−4.53
Left middle occipital gyrus	74	−44.6	−75.7	37.4	−3.58

*Note*: Coordinates are presented in MNI space and represent the center of mass of each cluster.

To aid in the interpretation of these findings, we explored whether the regions detected in Model 4 (Figure 2b) were regions that were implicated in the main effect of SS‐Go (Figure 2a). Of the 2401 voxels detected in Model 4, 651 (27%) overlapped with voxels that showed a significant, positive main effect of SS‐Go. Given that 30% (75,538/252,673) of all voxels in the mask were associated with a significant, positive main effect of SS‐Go, there is less overlap in these analyses than would be expected by chance (*Χ* ^2^ = 8.79, *p* < .003). A visualization of the overlap of these effects overlaid on a brain anatomical image is provided in Figure S1. Table S2 identifies the locations of overlapping clusters containing at least 20 voxels (an arbitrary threshold to exclude the large number of very small, likely non‐meaningful clusters of overlap).

### 3.4 Network fMRI analyses

**FIGURE 3:**
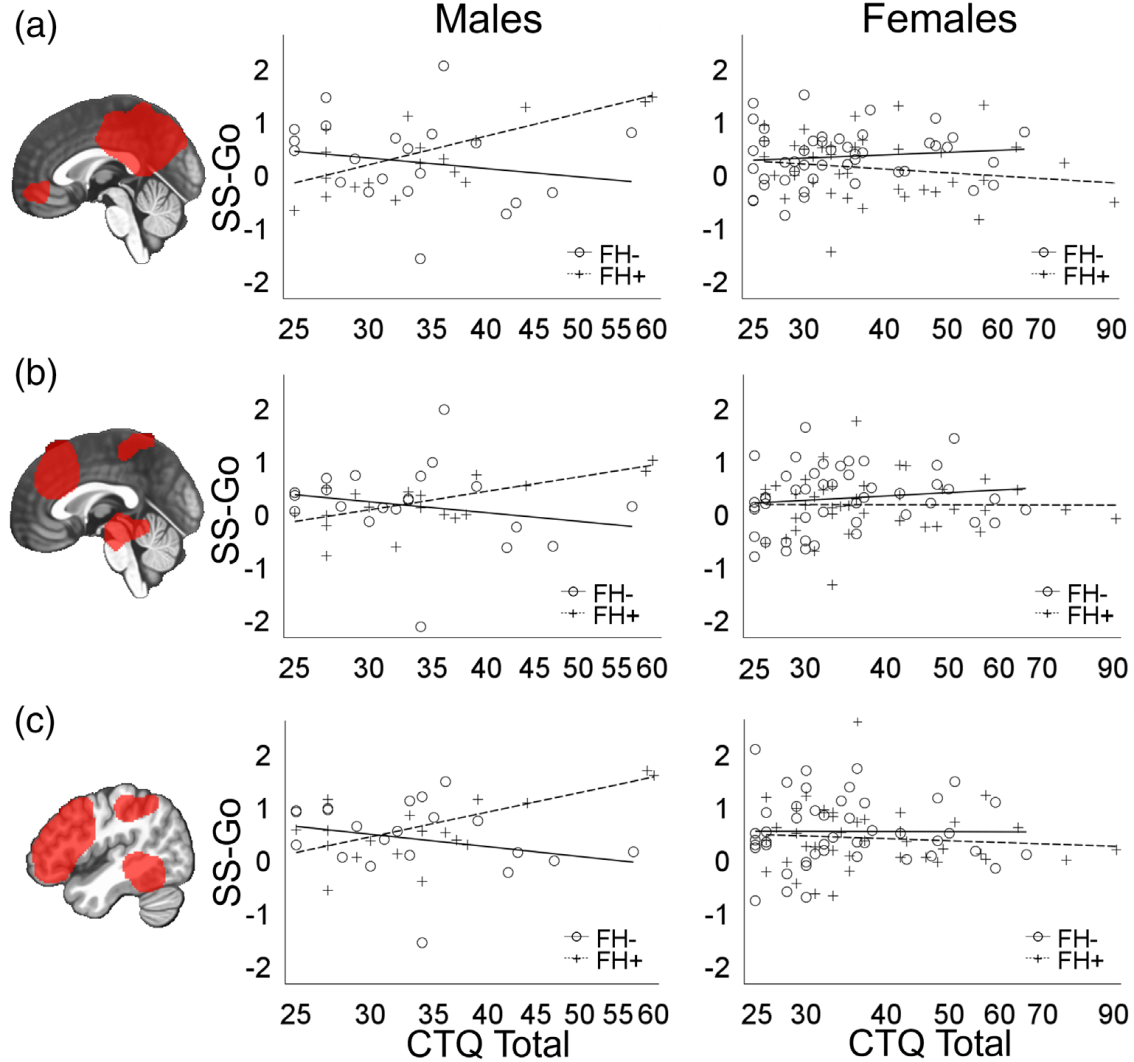
Network‐level neuroimaging results. Scatter plots of the effects of Childhood Trauma Questionnaire (CTQ) total scores on the contrast between successful stop trials and go trials (SS‐Go) for the (a) default‐mode network (b) executive control network, and (c) left frontoparietal network by family history density (FH) and sex. For visualization purposes, FH was binarized such that subjects with a family history density ≤0.25 were designated as FH‐ and >0.25 was designated as FH+.

Of the 10 networks tested, three‐way interaction effects of FH, CM, and sex were detected for three networks: the default‐mode network (Figure 3a; *t* = −3.03, *p* = .003), executive control network (Figure 3b; *t* = −2.94, *p* = .004), and left frontoparietal network (Figure 3c; *t* = −2.76, *p* = .007). These effects survived FDR correction for multiple tests. The results from these analyses mirrored those in Figure 2b from the whole‐brain voxel‐wise analysis in which there were sex differences in the effects of CTQ scores on task‐related network activation depending on FH density. Analyses in the Supplemental Materials show that these findings are not confounded by their effects on task performance or substance use.

### 3.5 Brain network relationships with behavioral symptoms

The canonical correlation analysis revealed two significant correlations between brain network and psychiatric measures (canonical correlation 1 = 0.43, *p* = .002; canonical correlation 2 = 0.39, *p* = .022). To aid in interpreting the canonical correlations, the correlations between the raw variables for the brain and the canonical variates for the brain as well as correlations between the raw variables for psychiatric measures and the canonical variates for psychiatric measures are presented in Table 4. The first canonical correlation indicated that greater activation of the executive control and left frontoparietal networks correlated with general reductions in psychopathology across ADHD, anxiety, depression, and substance use domains. A second canonical correlation indicated that increased default‐mode network activation, coupled with activation of executive control and left frontoparietal networks, correlated with greater psychological resilience and reduced ADHD and depressive symptoms, but also with heightened anxiety.

**TABLE 4:** Variable correlations with canonical variates for network activation and behavior

		Canonical correlation 1	Canonical correlation 2
Brain variables	Network 4 (DMN)	−0.09	**0.99**
	Network 8 (ECN)	**0.39**	**0.79**
	Network 10 (LFPN)	**0.70**	**0.71**
Psychiatric variables	Audit score	**−0.22**	0.02
	Cannabis use	**−0.46**	**−0.22**
	Tobacco use	**−0.63**	**0.28**
	Inattentive symptoms	**−0.63**	**−0.51**
	Hyperactive–impulsive symptoms	**−0.43**	**−0.48**
	Depression symptoms	**−0.30**	**−0.48**
	Trait anxiety	**−0.29**	**0.41**
	Resilience	−0.10	**0.53**

*Note*: Red and blue colors represent the strength of positive and negative correlation values, respectively, and are provided to enhance visualization of brain‐behavioral relationships. Significant (*p* < 0.05, uncorrected) correlations between raw variables and their corresponding canonical variates are indicated with bold font.

Abbreviations: DMN, default‐mode network; ECN, executive control network; LFPN, left frontoparietal network.

Pairwise Pearson correlations, covarying for the same variables as the canonical correlation analysis, are reported in Table S3. The uncorrected results for these univariate analyses largely mirror results of the multivariate correlation approach. Patterns of brain‐behavioral relationships were generally consistent across both sexes (Table S3), although significant relationships tended to be stronger in male subjects, with the exception of anxiety symptoms, which was stronger in females.

## 4 DISCUSSION

Although we predicted the interaction of FH and CM would be associated with supra‐additive adverse effects on behavioral and neural measures of response inhibition, analyses revealed potential positive adaptations associated with greater risk exposure that were sex‐dependent. Effects of risk factors were more pronounced in male subjects, in which CM was associated with heightened impulsivity and reduced BOLD response in males without FH; however, males with higher levels of both risk factors were more likely to exhibit better behavioral performance and greater BOLD response during response inhibition. Effects in females were overall smaller but trended in the opposite direction as males. Additionally, whereas greater BOLD responses in the executive control network and left frontoparietal network during the stop‐signal task was associated with reduced symptoms of ADHD and depression, BOLD responses in the default‐mode network was associated with both increased trait anxiety and higher resilience scores. The findings suggest the potential for sex‐specific neurobehavioral adaptations in response to risk and indicate brain mechanisms by which males and females may differ in the prevalence and expression of different forms of psychopathology.

### 4.1 Behavioral analysis

Prior studies have pointed to poor response inhibition in individuals with FH (Acheson et al., 2011; Dougherty et al., 2014) and childhood adversity (Mueller et al., 2010), although behavioral effects are not consistently found (e.g., Elton et al., 2014; Khemiri et al., 2020). Our results highlight the complexity of effects of risk factors and sex on SSRT. Specifically, the data indicate males with increased levels of both heritable and environmental risk for AUD demonstrate a more “resilient” behavioral phenotype (i.e., preserved SSRT) compared with males with only moderate levels of risk, whereas minimal effects were seen in females (Figure 1). The observed interaction effect mirrors previous findings in at‐risk young adult alcohol drinkers who were otherwise healthy, which showed a significant sex‐by‐FH interaction in which males with familial risk had improved inhibitory control relative to at‐risk females (Weafer et al., 2015). An interpretation of these findings is that high‐risk males who do not meet criteria for any psychiatric disorders at 18–19 years of age tend to exhibit positive adaptations such as preserved response inhibition. On the other hand, this behavioral measure may not be as strong of an index of AUD‐related risk or resilience in females. The inclusion of a potentially large number of individuals expressing heightened resilience in this sample may partly account for the absence of main effects of risk factors on SSRT.

### 4.2 Task activation

Analyses detected risk‐ and sex‐dependent increases and decreases in BOLD response across the cortex in regions associated with the left frontoparietal network, executive control network, and default‐mode network. Greater BOLD response during response inhibition has been interpreted as reflecting either compensatory adaptations or negative consequences (Morein‐Zamir et al., 2015). Previous studies have demonstrated that, prior to significant substance use, at‐risk youth with FH exhibit reduced prefrontal activation during response inhibition, particularly in the dorsal anterior cingulate cortex and lateral prefrontal cortex (Hardee et al., 2014; Schweinsburg et al., 2004). However, there are also data supporting greater activation of the bilateral middle frontal gyrus and rostral anterior cingulate in adolescents with FH during Stroop interference (Silveri et al., 2011). Reduced BOLD response during response inhibition in adolescence has been associated with future heavy alcohol use (Norman et al., 2011), supporting the conclusion that enhanced BOLD response is generally protective. The association of greater activation of the executive control network and left frontoparietal network with lower trajectories of alcohol use (Table 4) further supports this conclusion. Thus, increases or decreases in BOLD response during response inhibition may partly reflect individual differences in vulnerability or resilience.

Although there was partial overlap between the main effect of response inhibition (SS‐Go) and sex‐dependent effects of risk factors (Model 4), particularly in lateral prefrontal regions (Figure S1 and Table S2), the detected sex‐dependent effects of risk factors were largely in regions not typically considered key regions for inhibitory control (Congdon et al., 2010). For example, the anterior medial prefrontal cortex and superior frontal gyrus, posterior cingulate gyrus, and portions of the left inferior frontal gyrus were unique to the Model 4 interaction effect. This dissociation suggests that the sex‐dependent brain representation of risk or resilience during response inhibition is primarily reflected in the level of engagement of secondary—potentially compensatory—brain processes. More work is needed to understand the role of these supplementary brain regions during response inhibition in the context of risk for AUD.

In the current study, AUD risk was associated with alterations in the left frontoparietal network including the left inferior frontal gyrus. Consistent with the current findings, a previous neuroimaging study in males and females with or without FH reported enhanced recruitment of the left inferior frontal gyrus during response inhibition in at‐risk individuals (DeVito et al., 2013). In both that study and the current study, the effect was driven by males, with high‐risk males demonstrating enhanced recruitment of the left inferior frontal gyrus. The current study adds further evidence that effects on this region are driven by a combination of both heritable and environmental risk factors. Similarly, a study in at‐risk adults with histories of childhood maltreatment indicated sex differences in left‐versus‐right‐lateralized functional connectivity during a stop‐signal task and corresponding sex differences in the connectivity relationships to SSRT and ADHD symptoms. Response inhibition tends to rely more on the right inferior frontal gyrus (Aron et al., 2003), and greater recruitment of the left inferior frontal gyrus has been interpreted as compensatory (DeVito et al., 2013). Indeed, we observed that males with higher levels of both heritable and environmental risk also exhibited greater activation of the left frontoparietal network and preserved SSRTs, consistent with these brain changes representing risk‐related compensatory adaptations.

The executive control network, including two clusters in the medial prefrontal cortex, also demonstrated a significant interaction of FH, CM, and sex. The medial prefrontal/anterior cingulate region represents a major hub of an inhibitory control network that also include the bilateral anterior insula/inferior frontal gyrus (Elton et al., 2014). As noted above, a previous study demonstrated sex differences in the effects of childhood maltreatment on the functional connectivity between the anterior cingulate cortex and left and right anterior insula/inferior frontal gyrus, suggesting an important role of this brain region in sex‐by‐risk factor interactions on inhibitory control. In fact, the anterior cingulate cortex has been implicated in resilience measured with CD‐RISC (Brunetti et al., 2017; Long et al., 2019). The current study further suggests a role for this brain region in sex‐specific functional adaptations in response to both stress and heritable risk factors.

Our data also indicated increased (males with higher FH and higher CM) or decreased (males with either higher FH or higher CM) activation in regions associated with the default‐mode network (Figures 2b and 3), including medial prefrontal cortex and posterior cingulate cortex. Default mode network activity is typically suppressed during externally oriented tasks such as the stop‐signal task (Shulman et al., 1997), and its activation has been associated with poorer stop signal inhibition (Congdon et al., 2010). In fact, in the SS‐Go contrast for the whole sample (Figure 2a), default‐mode‐related regions generally showed non‐significant (e.g., posterior cingulate cortex) or negative (ventromedial prefrontal cortex) activation. However, the default‐mode network is involved in monitoring internally represented information (Spreng et al., 2009). Variations in task‐induced BOLD response in these regions related to risk factors and sex may reflect altered vigilance processes during task performance, an inference supported by the positive correlation of this network with anxiety symptoms (Tables 4 and S3). Furthermore, the finding that greater default‐network activation was also associated with higher resilience scores is consistent with the possibility that this effect represents a marker of resilience related to AUD risk factors.

Supplemental analyses that included SSRT as a covariate indicated that the effects of risk factors and sex on network activation were not driven by the differences in behavioral performance. Furthermore, other analyses suggested that brain effects were not related to subject‐level variation in prior substance use. This supports the contention that these brain changes are more closely associated with exposure to risk factors than to the behavioral consequences of those risk factors.

### 4.3 Brain relationships to psychiatric symptoms

Consistent with the notion that lesser activation during response inhibition is associated with greater risk for poor substance use outcomes, a correlation analysis linked lower SS‐Go contrast values within the executive control and left frontoparietal networks not only with increases in alcohol use over the follow‐up period, but also greater incidence of cannabis and tobacco use at follow‐up, greater symptoms of inattention and impulsivity, and greater depression (Table 4). However, note that the univariate correlation between SS‐Go contrast values and change in AUDIT scores was not significant (Table S3). On the other hand, greater default‐mode network engagement for SS‐Go was associated with higher resilience scores. Given sex differences in effects of risk factors on the BOLD response during response inhibition, these canonical correlations may reflect sex differences in the expression of psychopathology. For example, differing brain consequences and adaptations in males versus females could be responsible for different outcomes in response to stress and familial risk. Specifically, ADHD and other externalizing behaviors are more prevalent in males (Ramtekkar et al., 2010), whereas anxiety disorders are more prevalent in females (McLean et al., 2011). Thus, males and females in this study with greater levels of both heritable and environmental risk demonstrated sex‐specific brain adaptations that appear to be advantageous in reducing particular negative outcomes. Importantly, such adaptations do not preclude the possibility that these individuals could have poor outcomes in the future, particularly with regards to alcohol and other substance use. In particular, certain brain adaptations in response to stress are proposed to be developmentally adaptive, despite producing vulnerabilities to poor mental health outcomes later in life (Teicher et al., 2016).

### 4.4 Sex differences

Biological sex is an important moderator of risk and expression for various mental disorders, likely due to some combination of sex differences in genetics, hormones, and life experiences (Rutter et al., 2003). The males and females in this study did not demonstrate baseline differences in mental health and substance use measures but did differ in reported sexual abuse. However, the sexual abuse subscale of the CTQ only accounts for a small proportion of variance in the total CTQ scores due to the generally low scores in both sexes and are thus unlikely to fully account for the observed sex effects during the stop‐signal task. Although the mechanisms for sex differences in the current study remain speculative, there are notable sex differences in the brain that could account for the observed findings. Numerous sexual dimorphisms in the healthy brain have been reported (Chen et al., 2007; Goldstein et al., 2001; Maller et al., 2006; Zaidi, 2010). In particular, males have greater volume in the anterior cingulate cortex (Liu et al., 2020), which is a key region involved inhibitory control and also demonstrated sex differences in the current analysis. Although the developmental distribution of estrogen receptors may account for structural differences in the brain (Goldstein et al., 2001; Liu et al., 2020), there are also sex differences in brain function, perhaps related to circulating sex steroids (Dreher et al., 2007; Smith et al., 2014). For example, during a stop‐signal task, males exhibited increased activation of the anterior cingulate cortex and superior frontal gyrus (C‐sR et al., 2006), regions implicated in the current study, and females generally exhibit poorer behavioral control (Weafer et al., 2015). CM also differentially affects the neuroendocrine stress response in females compared with males (Bale & Epperson, 2015; DeSantis et al., 2011), which could account for sex differences in brain function related to CM observed in this study. Sex further modulates FH effects on the brain, such that males with FH exhibit enhanced activation of the left inferior frontal gyrus during response inhibition (DeVito et al., 2013). Thus, differences between the sexes in the effects of risk factors on brain regions involved in inhibitory control may arise from some combination of sex differences in brain structure and function and sex differences in neuroendocrine and neurodevelopmental responses to risk.

### 4.5 Limitations

Analyses of task‐induced BOLD responses generally provide robust predictors of behavioral and psychiatric variables. Although this sample was relatively large, and analyses examined effects of continuous variables to enhance power of statistical analyses, the results will need to be reproduced in a larger sample. The results of this study likely reflect certain features of the study design and sample, such as the exclusion of psychiatric disorders and the inclusion of 18‐19‐year‐old first‐year college students. Thus, this sample likely includes more resilient individuals than a general community sample. Additionally, family history was measured by self‐report assessments, and many subjects may have had limited knowledge of AUD in their extended family. Finally, continued longitudinal assessments, which are being collected on this sample, will be needed to determine whether effects of risk factors on the brain predict future trajectories of alcohol use and other mental health variables into adulthood.

## 5 CONCLUSIONS

Family history of AUD and childhood maltreatment had sex‐dependent interacting effects on brain function during a stop‐signal task. Sex differences in brain responses to risk factors may account for differential expression of psychopathology between the sexes. Efforts to tailor interventions based on risk factors and/or biological sex may be warranted. For example, these data suggest that AUD treatments targeting left prefrontal functioning may have stronger advantages in males.

## CONFLICT OF INTEREST

The authors declare no conflict of interest.

## Supporting information


**DATA S1.** Supporting Information

## Data Availability

The data that support the findings of this study are available from the corresponding author upon reasonable request.
