# Supplementary material for: Sex moderates family history of alcohol use disorder and childhood maltreatment effects on an fMRI stop‐signal task
Source: Hum Brain Mapp. 2023 Feb 1;44(6):2436–50. doi: 10.1002/hbm.26221 (PMC10028663; doi:10.1002/hbm.26221)
Supplement: Supplementary file 1 — DATA S1. Supporting Information [file HBM-44-2436-s001.pdf]

Behavioral Data

Supplemental Table 1. Pearson Correlations between Risk Factors and Stop-Signal Reaction Time.

		FH	CM
**All**	r	0.15	-0.08
	p	*0.10*	*0.39*
**Males**	r	0.18	0.03
	p	*0.29*	*0.84*
**Females**	r	0.13	-0.14
	p	*0.24*	*0.20*

FH, family history density; CM, childhood maltreatment measured as log-transformed Childhood Trauma Questionnaire total scores.

Voxel-wise fMRI Activation Analysis

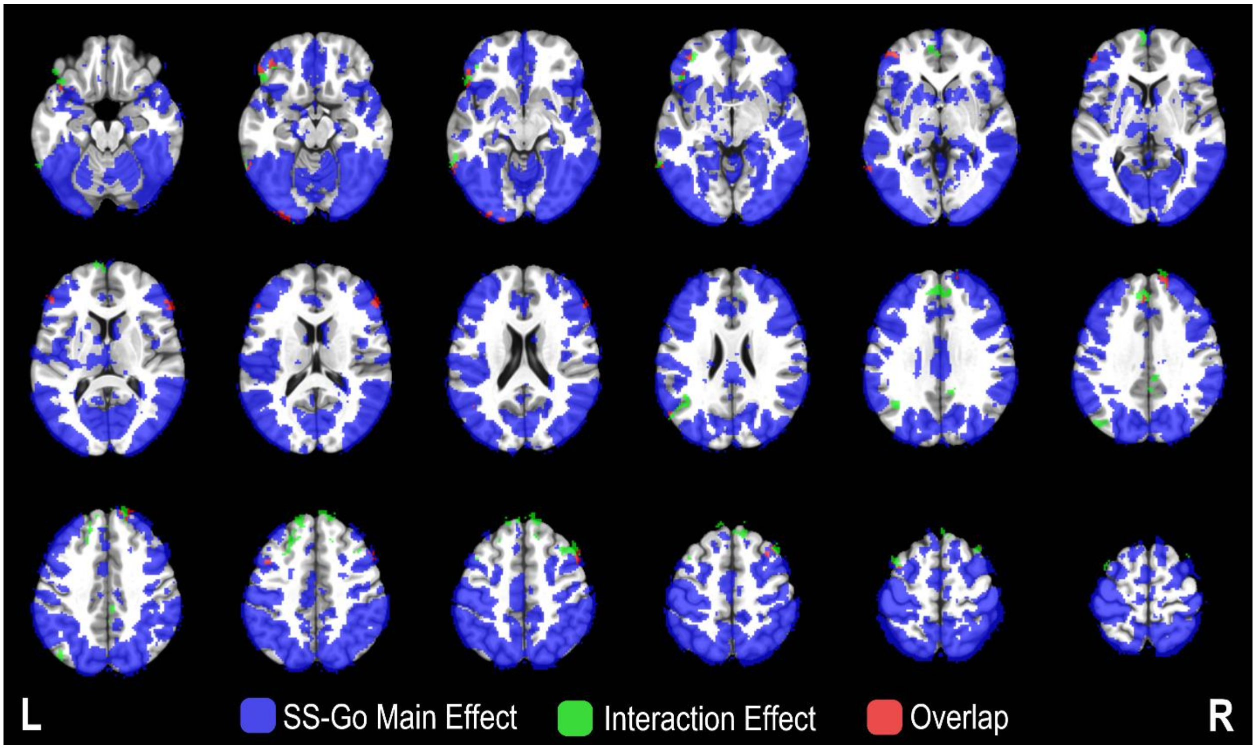

Supplemental Figure 1. The overlap of the main effect of the Successful Stop minus Go contrast (SS-Go) with the interacting effects of family history, childhood maltreatment, and sex detected in Model 4.

Supplemental Table 2. Overlapping Effects of the Interaction of Family History, Childhood Maltreatment, and Sex and the Main Effect of Successful Stops minus Go Trials

Brain Region	n voxels	x	y	z
Right Inferior Frontal Gyrus (*Pars Triangularis*)	104	56.7	34.5	13.6
Left Middle Frontal Gyrus	103	-48.2	44.6	3.6
Left Occipital Pole	84	-29.4	-99.7	-13.4
Left Inferior Frontal Gyrus (*Pars Orbitalis*)	78	-46.2	36.3	-13
Right Superior Frontal Gyrus	67	15.5	56.7	35.4
Left Inferior Temporal Gyrus	50	-63.9	-53.7	-8.7
Right Middle Frontal Gyrus	31	50.8	16	48.3
Left Angular Gyrus	20	-53.4	-61.1	22.3

Only clusters positively associated with the main effect of the contrast of successful stops minus Go trials are included. Interaction effects of risk factors and sex are all negative. Coordinates are presented in MNI space and represent the center of mass of each cluster. Results displayed in this table are restricted to clusters containing at least 20 voxels.

Beta-Series Correlation of Large-Scale Networks

Given previously-reported interacting effects of CTQ scores and sex on functional connectivity during a stop-signal task[33], we performed a beta-series functional connectivity analysis[63] to examine effects of risk factors and sex on functional interactions between large-scale networks. First, we reran the AFNI GLM analyses, modeling each successful stop and each Go trial separately. Beta estimates for each trial for both of these trial types were calculated in a voxel-wise manner across the whole brain, and the mean value was subsequently extracted within each binary network mask. This produced a beta series for successful stops and a beta series for Go trials for each network. We focused our functional connectivity analysis on the three networks that were significant for the network-level task activation analysis described in the main text (i.e., default-mode network, executive control network, and left frontoparietal network). We calculated the correlations among these three networks during successful stops and Go trials separately, representing network-level functional connectivity during each trial type. To compare functional connectivity during successful stops with Go trials, a SS-Go contrast was computed as the difference in Fisher-z transformed correlations for SS and Go trials for each pair of networks. There were no significant main or interaction effects of FH, CM, and sex on task-dependent

functional connectivity between these networks, even at an uncorrected $p<0.05$.

Network fMRI Analyses tests for Confounding Variables

We next explored whether effects of risk factors on network activation are confounded by their effects on task performance or substance use. First, we controlled for SSRT by including it as a predictor in the linear regression model testing effects of FH, CM, and sex on network activation. Three-way interaction effects of FH, CM, and sex remained significant for the default-mode network ($t=-2.68$, $p=0.008$), executive control network ($t=-3.01$, $p=0.003$), and left frontoparietal network ($t=-3.41$, $p<0.001$). We also tested these effects when controlling for substance use, represented by AUDIT total score, cannabis use (yes/no), and tobacco use (yes/no). Again, three-way interaction effects remained significant for the default-mode network ($t=-3.29$, $p=0.001$), executive control network ($t=-2.82$, $p=0.006$), and left frontoparietal network ($t=-3.12$, $p=0.002$).

Supplemental Table 3. Pairwise Correlations between Brain Networks and Psychiatric Symptoms

	ALL			MALES			FEMALES		
	Network 4 (DMN)	Network 8 (ECN)	Network 10 (LFPN)	Network 4 (DMN)	Network 8 (ECN)	Network 10 (LFPN)	Network 4 (DMN)	Network 8 (ECN)	Network 10 (LFPN)
**Δ AUDIT Score**	0.00	-0.04	-0.06	-0.09	0.04	0.17	-0.01	-0.07	-0.12
	0.99	*0.71*	*0.58*	*0.65*	*0.85*	*0.41*	*0.93*	*0.60*	*0.33*
**Cannabis Use**	-0.04	-0.06	-0.19	0.01	-0.18	-0.27	-0.07	-0.09	-0.22
	0.71	*0.53*	*0.07*	*0.95*	*0.39*	*0.18*	*0.55*	*0.49*	*0.08*
**Tobacco Use**	0.11	-0.09	-0.12	-0.09	-0.27	-0.33	0.22	0.05	0.00
	0.30	*0.40*	*0.25*	*0.65*	*0.18*	*0.10*	*0.08*	*0.70*	*0.98*
**Inattentive Symptoms**	-0.20	**-0.35**	**-0.40**	-0.51	**-0.63**	**-0.60**	-0.06	-0.18	**-0.28**
	0.06	*<0.001*	*<0.001*	*0.01*	*0.00*	*0.00*	*0.65*	*0.15*	*0.02*
**Hyperactive-Impulsive Symptoms**	-0.18	**-0.22**	**-0.32**	-0.36	**-0.44**	**-0.42**	-0.07	-0.08	**-0.26**
	0.09	*0.03*	*0.001*	*0.07*	*0.03*	*0.03*	*0.59*	*0.51*	*0.03*
**Depression Symptoms**	-0.19	**-0.32**	**-0.28**	-0.28	**-0.46**	**-0.43**	-0.17	-0.21	-0.18
	0.07	*0.002*	*0.01*	*0.17*	*0.02*	*0.03*	*0.18*	*0.08*	*0.15*
**Trait Anxiety**	**0.20**	0.07	0.02	0.04	0.12	0.02	0.24	0.08	0.03
	0.05	*0.48*	*0.84*	*0.84*	*0.57*	*0.94*	*0.05*	*0.53*	*0.78*
**Resilience**	**0.24**	**0.24**	0.16	**0.48**	**0.51**	0.43	0.17	0.11	0.01
	0.02	*0.02*	*0.13*	*0.01*	*0.01*	*0.03*	*0.16*	*0.39*	*0.92*

Pearson correlations and p-values (*italics*) are reported for the pairwise correlation between brain networks and psychiatric symptoms. Δ AUDIT Score respresents change in AUDIT score from baseline to the one-year follow-up. Cannabis and tobacco use variables included here are from the one-year follow-up. **Bold** font indicates p<0.05. DMN, default-mode network; ECN, executive control network; LFPN, left frontoparietal network
